# A Treatise on Orthopedic Surgery

**Published:** 1890-07

**Authors:** 


**A Treatise on Orthopedic Surgery.** By EDWARD H. BRADFORD, M. D., Surgeon to the Children's Hospital, Boston City Hospital, and Samaritan Hospital; Instructor in Clinical Surgery, Harvard Medical School; and ROBERT W. LOVETT, M D., Surgeon to the Samaritan Hospital; Assistant Out-Patient Surgeon to the Children's Hospital; Out-Patient Surgeon to the Carney Hospital; formerly Assistant Surgeon to the New York Orthopedic Dispensary and Hospital. Illustrated with 789 wood engravings. Imperial octavo, pp. viii.—783. New York: William Wood & Company. 1890.

In the preface of this book the authors say that previous works on this subject have usually confined themselves to a consideration of the treatment of existing deformities, such as club-foot, lateral curvature, and bowlegs; that the prevention of deformity as well as its cure ought to be considered in such treatises; and that, for these reasons, diseases of the joints have been by them considered at considerable length. This is offering at once a good and sufficient reason for the appearance of this book.

Orthopedic surgery has grown into a distant specialty; separate chairs are created to teach it in the medical colleges; and separate medical societies, local and national, are organized to discuss its various and important phases. There is no more important duty that a physician can perform than to cure the deformity of a child that else would go through life a cripple, become, perhaps, dependent upon charity —certainly upon others—and besides offend the delicate æsthetism that should ever apply to the artistic proportions of the human body as elsewhere.

All honor, therefore, to these authors, as well as to the erudite and accomplished Gibney, and his colleagues in New York, who are doing such noble and scientific work. In all the cities of the land where the orthopedic surgeon can now be found, he should be sustained by his professional brethren, and aided in his good works.

This book is by far the most complete and interesting treatise that has yet appeared on orthopedy, and it will undoubtedly quickly pass through its first edition. The illustrations are profuse and clear, and the general execution of the book is in keeping with the excellent work turned out by Messrs. William Wood & Company, and is the second volume of the Specialties in the Practice of Medicine series.

